# A Colorimetric Microplate Assay for DNA-Binding Activity of His-Tagged MutS Protein

**DOI:** 10.1007/s12033-016-9949-7

**Published:** 2016-05-30

**Authors:** Michał Banasik, Paweł Sachadyn

**Affiliations:** Department of Molecular Biotechnology and Microbiology, Gdańsk University of Technology, ul. Narutowicza 11/12, 80-233 Gdańsk, Poland

**Keywords:** DNA–protein binding, MutS, DNA mismatch, Nickel-coated microplate, His-tag

## Abstract

A simple microplate method was designed for rapid testing DNA-binding activity of proteins. The principle of the assay involves binding of tested DNA by his-tagged protein immobilized on a nickel-coated ELISA plate, following colorimetric detection of biotinylated DNA with avidin conjugated to horseradish peroxidase. The method was used to compare DNA mismatch binding activities of MutS proteins from three bacterial species. The assay required relatively low amounts of tested protein (approximately 0.5–10 pmol) and DNA (0.1–10 pmol) and a relatively short time of analysis (up to 60 min). The method is very simple to apply and convenient to test different buffer conditions of DNA–protein binding. Sensitive colorimetric detection enables naked eye observations and quantitation with an ELISA reader. The performance of the assay, which we believe is a distinguishing trait of the method, is based on two strong and specific molecular interactions: binding of a his-tagged protein to a nickel-coated microplate and binding of biotinylated DNA to avidin. In the reported experiments, the solution was used to optimize the conditions for DNA mismatch binding by MutS protein; however, the approach could be implemented to test nucleic acids interactions with any protein of interest.

## Introduction

DNA–protein interactions are critical for as fundamental biological processes as DNA replication, transcription, DNA recombination and repair. A number of methods addressing the cardinal problem of detecting DNA–protein complexes have been developed. Gel retardation [1, 2] or nitrocellulose filter binding assays [3] are the examples of simple, but rather time-demanding, approaches. The advanced methods such as surface plasmon resonance [4], laser scattering or atomic force microscopy [5] open more possibilities but they depend on sophisticated instruments.

Mismatch binding proteins known as MutS in prokaryotic and MSH in eukaryotic cells act as the guards of DNA replication fidelity [6]. MutS is the first element of DNA mismatch repair system found in most but not all prokaryotic organisms [7]. MutS recognizes a mispaired base in DNA, which triggers a sequence of events resulting in mismatch correction. A number of methods employing MutS for DNA mismatch detection have been developed [8–11]. Here we present a microplate assay that could be a convenient solution for the preliminary detection of DNA mismatch binding activity.

The method reported in this study has been designed in order to examine DNA binding to proteins immobilized on nickel-coated ELISA plates through the use of oligohistidine tags. The immobilized protein captures the tested biotin-labelled DNA fragments that are detected by avidin conjugated to horseradish peroxidase which catalyses a colorimetric reaction.

In the experiments presented herein, we implemented a solution to optimize the conditions for DNA mismatch binding by MutS proteins. However, this approach could be useful to examine DNA-binding properties of different proteins of interest.

## Materials and Methods

### Preparation of Tested DNA Fragments

The DNA fragments were prepared from synthetic oligonucleotides. The biotinylated oligonucleotide T49, BIOT-ACAGATCCACTGTGCGACGAGCTGTGCCGCACGGTGATCGCAGCCGCTG was mixed with an equimolar amount of fully complementary A49, CAGCGGCTGCGATCACCGTGCGGCACAGCTCGTCGCACAGTGGATCTGT or almost fully complementary G49 CAGCGGCTGCGATCACCGTGCGGCGCAGCTCGTCGCACAGTGGATCTGT in order to obtain fully complementary or mismatched DNA fragments, respectively. To prepare oligonucleotide duplexes, the mixtures of HPLC purified oligonucleotides were suspended in a buffer containing 10 mM Tris–HCl (pH 8.8/25 °C), 50 mM KCl, 0.08 % (v/v) Nonidet P40 and 5 mM MgCl_2_ in a final volume of 100 μl to a final concentration of 10 μM, and subjected to heating and cooling (92 °C/120 s, 65 °C/120 s, 25 °C/120 s).

### Protein Purification

The recombinant MutS proteins were produced using *Escherichia coli* BL21(DE3)pLysS transformed with recombinant plasmid carrying the cloned *mutS* genes from *Deinococcus radiodurans* (DrMutS), *Escherichia coli* (EcMutS) and *Thermus thermophilus* (TthMutS**)**, and purified using immobilized metal ion affinity chromatography (IMAC). Bacterial cell pellets were suspended in buffer A (50 mM phosphate buffer, pH 8.0/25 °C; 500 mM NaCl; 10 % glycerol (v/v)) and disrupted by sonication on ice. Total lysate was centrifuged and the supernatant was collected and applied to cOmplete his-tag purification resin containing immobilized Ni^2+^ ions (Roche, cat. no. 05893682001) equilibrated with buffer A. The column was washed with buffer A, following washing with buffer A supplemented with 10 mM imidazole. The his-tagged MutS protein was eluted with ten 1 ml portions of elution buffer A supplemented with 500 mM imidazole, following dialysis against buffer S (50 mM phosphate buffer, pH 8.0/25 °C; 200 mM NaCl; 10 % glycerol (v/v)) and concentration by centrifugation using ultrafiltration device with a 100 kDa cut-off (Viva Science, cat. no. Z614661) to a final volume of 500 µl. The proteins were diluted with 50 % (v/v) glycerol. Protein purity and concentration were determined by densitometric analysis of SDS-PAGE electropherograms.

### Protein Immobilization, DNA Mismatch Binding and Colorimetric Detection

The microplate wells (Pierce^®^ Nickel Coated Plates, (Thermoscientific, cat. no. 15442, lot #NH174556) were unblocked by washing with five 200 μl portions of washing/binding buffer WB (1 × PBS buffer (Sigma, cat. no 79383, lot. BCBF0586) supplemented with 1 % of Tween-20 (Cat. No P7949, lot #SZ2B2070V) and 5 mM MgCl_2_. One µg of his-tagged MutS protein was loaded per one microplate well in 100 μl of the same buffer. After 15 min of incubation, the well was emptied, following washing with five 200 μl portions of WB buffer and immediate loading of the tested biotinylated DNA (10 pmol) in 100 μl of WB buffer. After 10 min of incubation, the excess of unbound DNA was removed by washing with five 200 μl portions of WB buffer. DNA binding by the immobilized protein was detected with horseradish peroxidase conjugated to avidin (Extravidin, Sigma, cat. no. E2886, lot 098K4751). The well was loaded with 100 μl of Extravidin 1000 times diluted in WB buffer and after 10 min of incubation washed with five 200 μl portions of WB buffer, following immediate loading of 100 μl of TMB (3,3′,5,5′-Tetramethylbenzidine) chromogenic substrate solution (Sigma, cat. no T0440, lot 051M1850). The colour reaction was developed for 15 min, and the absorbance was measured with an ELISA reader (MultiSkan FC, ThermoScientific, cat. No. #51190000) at 620 nm. All steps of the protocol were carried out at room temperature.

### Determination of DNA Mismatch Binding Affinity and Specificity

The A_620_ absorbance values measured in the colour reaction were used to evaluate the affinity of MutS towards mismatched DNA and fully complementary DNA controls. The specificities were determined as the ratio of MutS affinity for mismatched to fully complementary DNA. In order to estimate background signals, the control experiments without DNA addition were performed. Statistical significance was determined with two-tailed heteroscedastic Student's t-test.

## Results

**Fig. 1:**
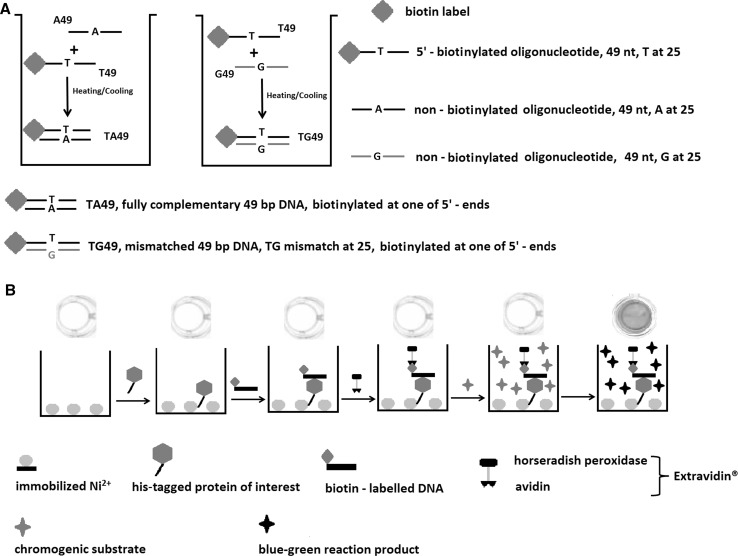
Principle of microplate colorimetric assay of DNA mismatch binding activity by his-tagged MutS. **a** preparation of mismatched and fully complementary DNA fragments; **b** protein immobilization, DNA mismatch binding and colorimetric detection

The principle of mismatch DNA-binding assay by his-tagged MutS immobilized on a nickel-coated microplate is presented in Fig. 1. In brief, the his-tagged MutS protein is loaded to microplate wells to become immobilized after short incubation. The wells are emptied and washed to remove the unbound protein. For protein–DNA binding, the examined, biotinylated DNA fragments are added to the wells. After short incubation, the wells are emptied and washed in order to remove the unbound DNA. The captured DNA is detected with horseradish peroxidase conjugated to avidin. Avidin binds biotin labels of captured DNA, while horseradish peroxidase catalyses the oxidation of TMB chromogenic substrate, thus developing blue-green colour. The results could be quantitated with an ELISA reader.

In order to examine mismatch DNA-binding properties of MutS, we compared the effects of capturing mismatched and fully complementary DNA. We designed an experimental setup where there are three microplate wells with immobilized his-tagged MutS. The mismatched DNA is added to the first well, while the fully complementary DNA and the loading buffer without DNA are loaded to the second and third control wells, respectively. MutS-DNA-binding affinity is estimated as the absorbances obtained in the colorimetric assay. The specificity towards the mismatch is calculated as the ratio of the signal for mismatched DNA to that for fully complementary DNA.

### DNA Mismatch Binding Affinity and Specificity at Different Binding Conditions

**Fig. 2:**
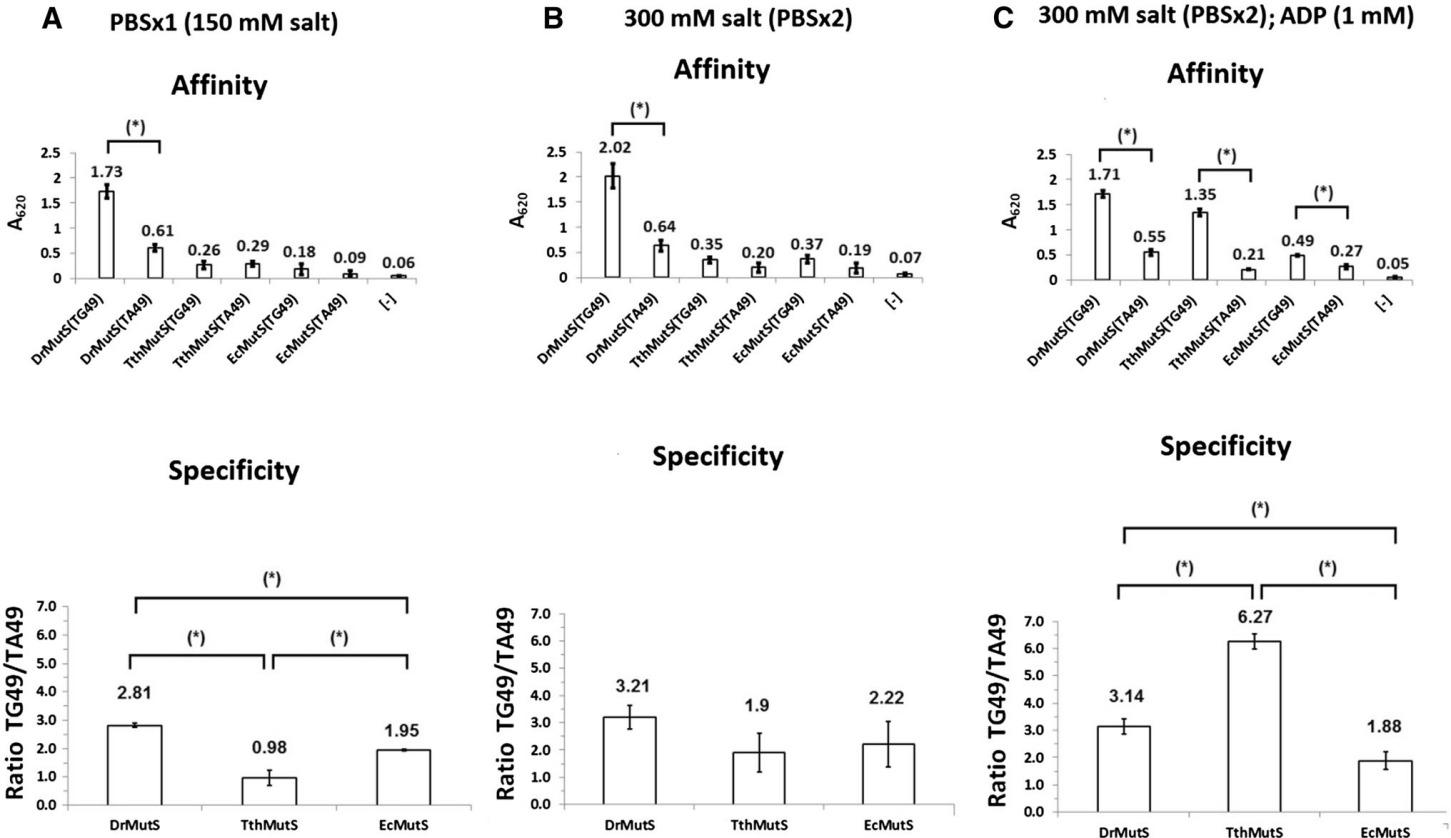
Impact of salt concentration and ADP on DNA mismatch binding by MutS proteins from three bacteria: *D.* *radiodurans* (DrMutS), *T. thermophilus (*TthMutS), and *E. coli* (EcMutS). The critical parameters of the experiments were as follows: **a** 150 mM salt concentration (PBSx1); **b** 300 mM salt concentration (PBSx2); **c** 1 mM ADP presence. The specificities were calculated as the ratio of affinities (absorbance values) for the mismatched (TG49) to fully complementary DNA (TA49). The background signal [−] is marked for comparison. Each experiment was repeated 3 times. Statistical significance was indicated with *asterisks* (*)

**Fig. 3:**
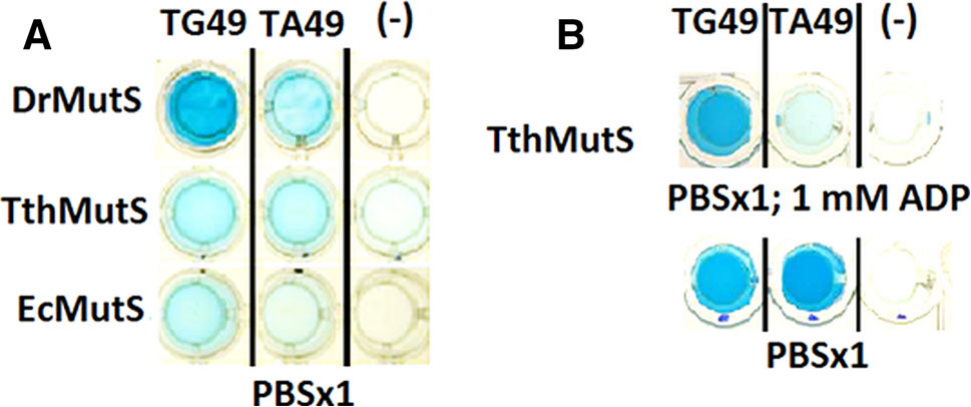
Colorimetric detection of DNA binding by his-tagged MutS immobilized on a nickel-coated ELISA plate. **a** binding of his-tagged MutS proteins from *D. radiodurans* (DrMutS)*, T. thermophilus* (TthMutS)*, and E. coli* (EcMutS) to mismatched (TG49) and fully complementary (TA49) DNA; **b** the impact of 1 mM ADP on MutS from *T. thermophilus*

In order to find the conditions where MutS proteins show high affinity for DNA mismatches and low non-specific binding to fully complementary DNA, we tested the impact of salt concentration and ADP presence in the buffer used for DNA binding and washing. We contrasted the results obtained for the buffer containing 150 mM salt (Figs. 2a, 3a) with those obtained for the buffer with doubled (300 mM) salt concentration (Fig. 2b), and for the 300 mM salt buffer with 1 mM ADP addition (Fig. 2c). We performed the experiments with three different his-tagged MutS proteins from *Escherichia coli*, *Deinococcus radiodurans*, and *Thermus thermophilus*. The proteins used in the experiments were purified from recombinant overproducing *E. coli* strains, and the his-tag tails were introduced as the translational fusions at their N-termini.

As a result, we were able to find that under 300 mM salt concentration the immobilized his-tagged MutS proteins exhibit both efficient binding of mismatched DNA and satisfactory specificity. Further, we observed that under the conditions of experiment, MutS from *D. radiodurans* showed a higher affinity towards mismatched DNA than those two others (Fig. 2a, b). In the presence of ADP, the specificity of MutS from *T. thermophilus* strongly increased (Fig. 3b) to exceed even that of MutS from *D. radiodurans* (Fig. 2c).

### Impact of DNA Concentration and Protein Amount

**Fig. 4:**
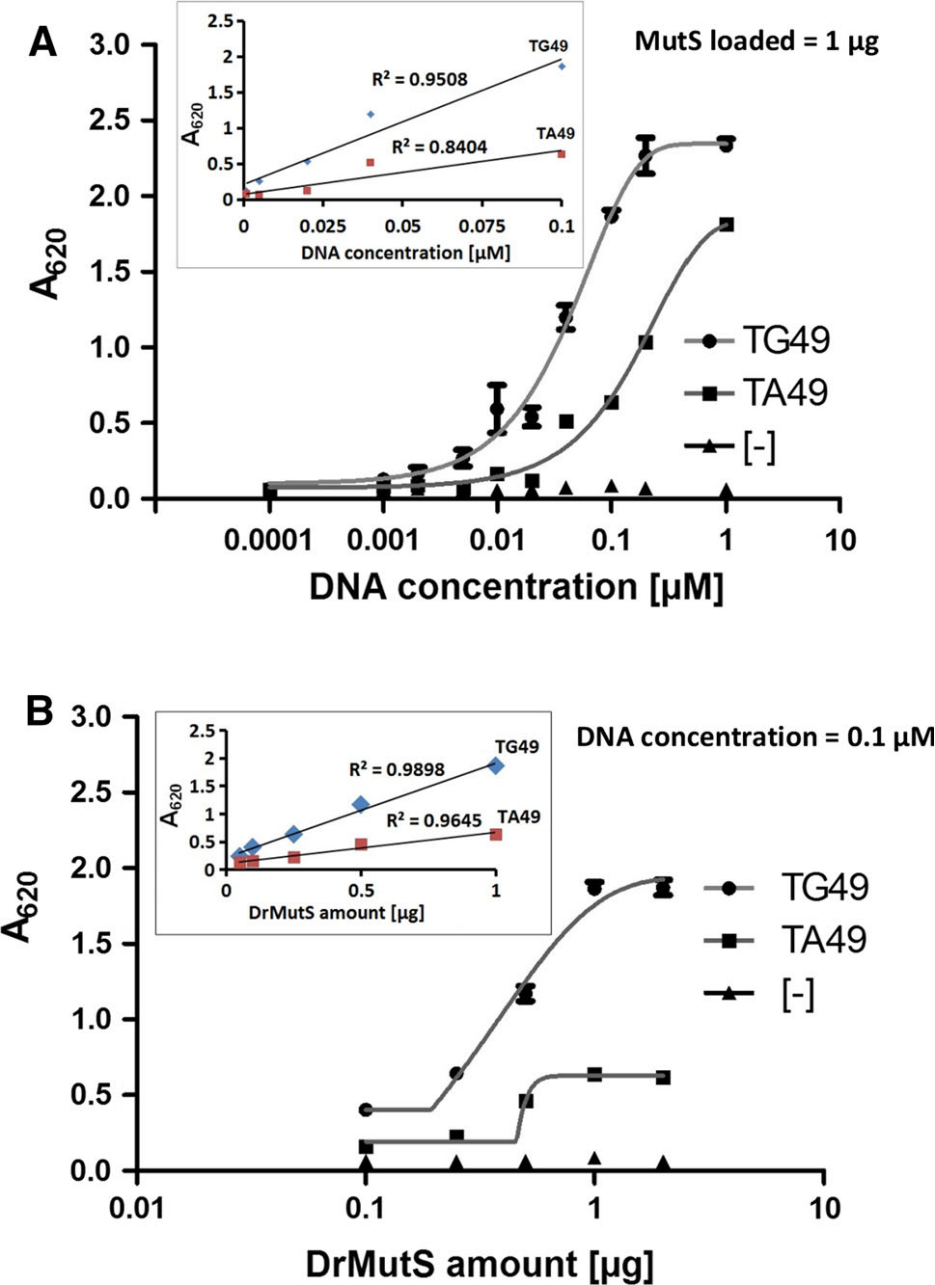
The impact of increasing amounts of DNA and MutS. The impact of increasing concentrations of DNA during binding with MutS (**a**) and amounts of loaded DrMutS (**b**) on the A_620_ absorbance. The ranges of linear analysis are shown in the *insets*. The specificities towards mismatched DNA are presented in Fig. 5

**Fig. 5:**
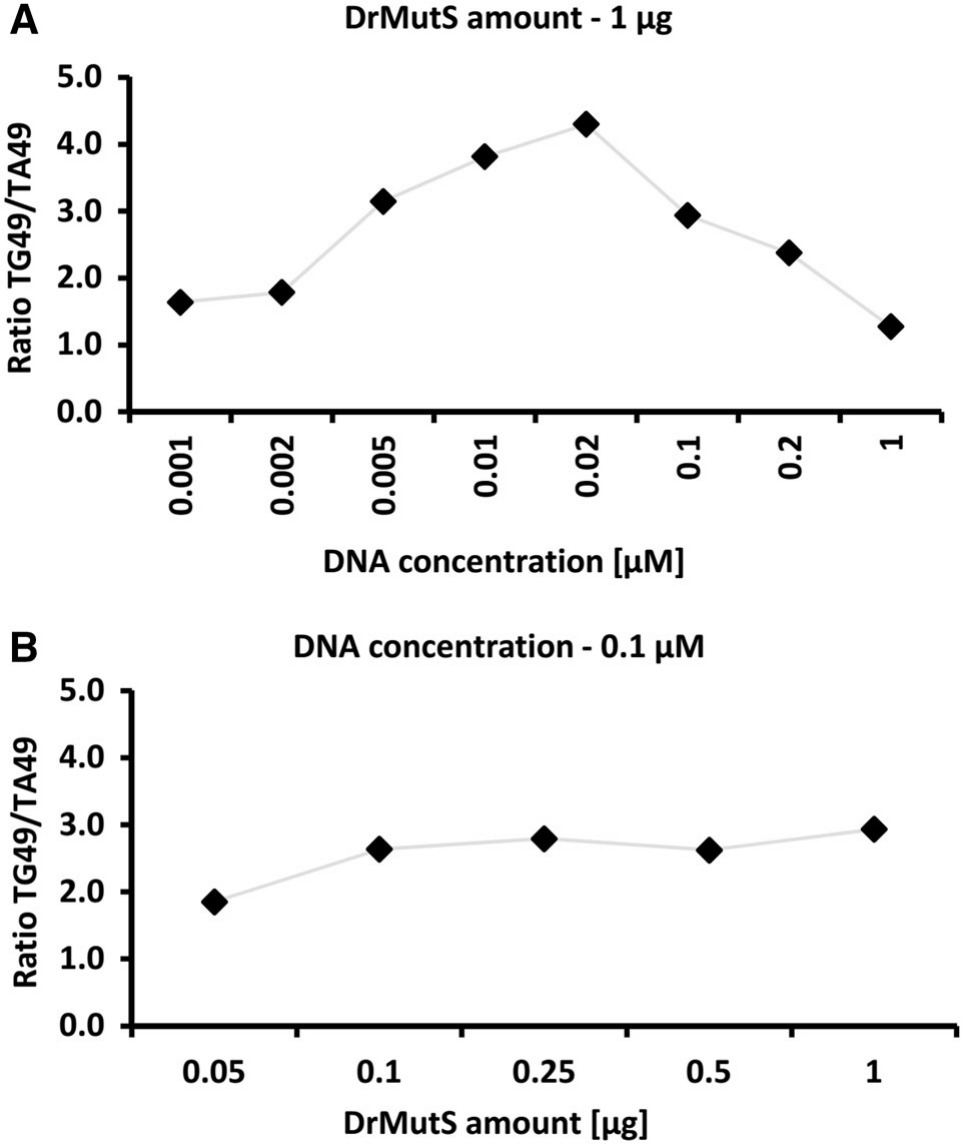
The effect of MutS and DNA amounts on the specificity towards DNA mismatches at different DNA concentrations (**a**) and amounts of DrMutS (**b**) loaded. The specificity is defined as the ratio of signals for the mismatched DNA to fully complementary DNA. The signal values (A_620_ absorbance) are indicated in Fig. 4

We performed two series of experiments in which either the amount of DrMutS protein added to the wells was kept constant (1 µg), while the DNA concentrations varied in the range of 0.0001–1.0 µM (Fig. 4a) or the concentration of loaded DNA was at the constant level (0.1 µM), whereas the amounts of loaded DrMutS varied from 0.05 to 2 µg (Fig. 4b). The obtained absorbance values were correlated with increasing amounts of either added DNA (Fig. 4a) or loaded MutS protein (Fig. 4b). The series of DNA-binding tests was performed in the buffer containing 150 mM salt without ADP addition, which corresponds to the conditions of the experiment presented in Fig. 2a. The measurements showed that the absorbance changed linearly with the increasing concentrations of loaded DNA within the range 0.001–0.1 µM (Fig. 4a, inset). Also, the results indicate that the working amounts of loaded his-tagged protein correspond to the range of 1–20 pmol (Fig. 4b, inset). A slight improvement in DNA mismatch binding specificity could be observed at lower amounts of loaded DNA (Fig. 5a). Changing the amounts of loaded MutS did not have an essential impact on specificity towards mismatched DNA (Fig. 5b). However, lowering DNA concentrations results in a decrease of colorimetric signal (Fig. 4a).

### Evaluation of Detection Sensitivity

**Fig. 6:**
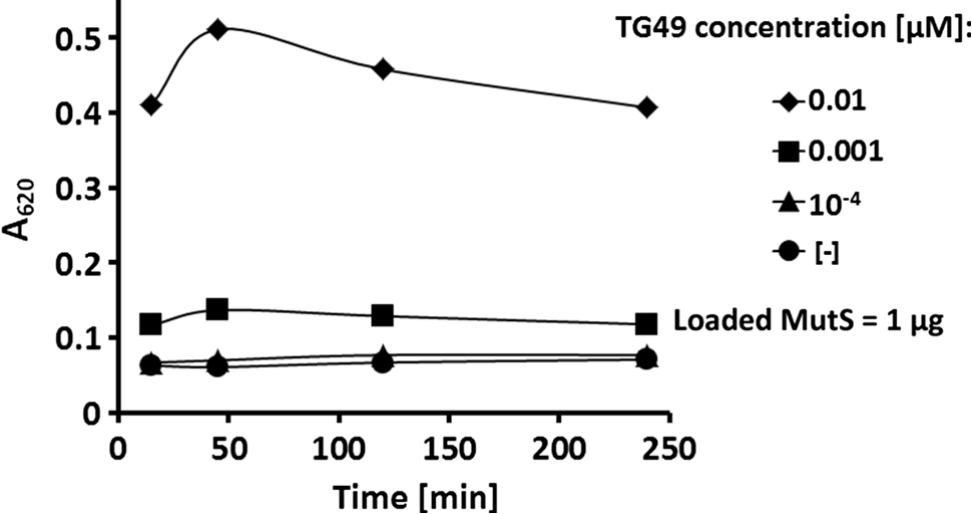
Prolonged time of colorimetric reaction does not enhance the sensitivity of DNA mismatch binding detection

The microplate assay with 1 µg of his-tagged-MutS loaded for immobilization was demonstrated to allow reliable detection of captured biotinylated DNA following binding in 100 µl volume at the concentrations ranging from 0.001 to 0.1 µM (Fig. 4a). In order to evaluate the limits of detection, the assay was performed for a series of decreasing DNA concentrations of mismatched DNA (0.01, 0.001, 0.0001 µM) and prolonged time of colorimetric reaction (Fig. 6). The results showed that the signal for 10^−4^ µM was close to the background level. In the case of DNA concentrations of 0.01 and 0.001 µM, the colorimetric signal could be easily discriminated from that of background. Nevertheless, we would recommend applying a higher working concentration of biotinylated DNA of 0.1 µM, where stronger signals are produced, as more useful to test different binding conditions (Fig. 2). Extending the time of colorimetric reaction from 15 to 245 min did not result in significant improvement of detection limit, while approximately a 25 % increase in signal was observed after a 45-min as compared to a 15-min reaction.

## Discussion

The microplate colorimetric assay we developed was useful to optimize DNA mismatch binding conditions and to compare the properties of his-tagged MutS proteins from three different bacteria: *T. thermophilus, E. coli, and D. radiodurans.*

We think that this approach could be implemented for testing interactions of nucleic acids with different proteins, on condition that an oligohistidine tag is added to the protein under examination and the nucleic acid substrate could be biotinylated. Other fusion tags than oligohistidine and other microplates than those nickel-coated, as well as other types of DNA labelling than biotin could be considered; however, we believe that the solutions we chose contributed to the assay performance. The advantages of the assay include:

- convenient protein immobilization on nickel-coated microplates;
- relatively low amounts of examined proteins—0.54–10.8 pmol per well (50 ng–1 µg of 92 kDa MutS monomer);
- relatively low amounts of tested DNA—0.1–10 pmol per well;
- low background signal owing to low binding solid phase of plastic microplates;
- non-radioactive labelling;
- relatively short time of experiment (up to 60 min)
- simple colorimetric detection—results could be observed with naked eye (Fig. 3) and quantitated with an ELISA reader.

Conditions such as ionic strength, pH, and the presence of cofactors or detergents could be easily modified, which facilitates testing of different factors influencing DNA–protein interactions. In contrast to gel retardation and filter binding assays, in this method, the protein–DNA interactions are examined in a buffer of choice. The necessity of adding the his-tag to the examined protein may be regarded as a complication; however, engineering an oligohistidine fusion domain is one of the preferred solutions used for rapid protein purification in molecular studies. The application of plastic solid phase allows low non-specific binding, but this is the addition of 1 % of Tween-20 which is a critical, and the absence of this reagent in the washing/binding buffer may result in a dramatic increase of background signal.

We designed the method for rapid assessment of protein–DNA binding. Considering the colorimetric signal changes linearly within some range of increasing DNA concentrations, the method allows relative quantitation of DNA-binding efficiencies. Nevertheless, we would not recommend using lower amounts of protein and DNA, as it may decrease detection sensitivity. Enzymatic colorimetric reaction enabled reliable detection of mismatched DNA binding at the concentrations of 0.01–0.1 μM. However, it should be underlined that we report the concentrations of loaded DNA, while the exact amounts of bound DNA are not determined in the assay.

## Concluding Remarks

A number of ELISA assays are broadly applied to examine protein-DNA interactions [9], including a few solutions reporting the use of nickel-coated microplates [12, 13]. The practical usefulness which distinguishes the assay we propose is based on two strong and specific molecular interactions: binding of his-tagged proteins to nickel-coated microplates and binding biotinylated DNA to avidin, as well as the sensitive detection with horseradish peroxidase. We recommend this approach for preliminary screening of DNA-binding proteins and for optimization of DNA–protein binding conditions.

## Abbreviations

T49: The biotinylated oligonucleotide, 49 bp in length, containing centrally located T in the position 25, complementary to A49 (within the full length of oligonucleotide) and G49 (complementary within the full length of oligonucleotide except for the position 25 where a TG mismatch is formed) (see below)
A49: The non-biotinylated oligonucleotide, 49 bp in length, containing centrally located A in the position 25
G49: The biotinylated oligonucleotide, 49 bp in length, containing centrally located G in the position 25
TG49: dsDNA, 49 bp in length, biotinylated at one 5′-end, containing a TG mismatch in the position 25
TA49: Fully complementary dsDNA, 49 bp in length, biotinylated at one 5′-end
